# Empathy, Autistic Tendencies, and Systemizing Tendencies—Relationships Between Standard Self-Report Measures

**DOI:** 10.3389/fpsyt.2019.00307

**Published:** 2019-05-10

**Authors:** Cornelia Sindermann, Andrew Cooper, Christian Montag

**Affiliations:** ^1^Department of Molecular Psychology, Institute of Psychology and Education, Ulm University, Ulm, Germany; ^2^Department of Psychology, Goldsmiths University of London, London, United Kingdom

**Keywords:** empathy, autism, systemizing, Interpersonal Reactivity Index, Empathy Quotient, Autism Spectrum Quotient, Systemizing Quotient–Revised

## Abstract

The aim of the present study was to investigate associations between four highly used self-report measures assessing empathy (measured as both a unidimensional and multidimensional construct), autistic tendencies, and systemizing tendencies. Participants in this study completed the following self-report measures: The Interpersonal Reactivity Index (IRI) and the Empathy Quotient (EQ) to measure empathy, and the Autism Spectrum Quotient (AQ) and the Systemizing Quotient–Revised (SQ-R) to assess autistic and systemizing tendencies, respectively. The final sample consisted of *N* = 1,098 participants (304 males) without a diagnosed autism spectrum disorder, most of whom were university students. The IRI scale “Perspective Taking” and the EQ were negatively related to the AQ in male and female participants, while the IRI scale “Empathic Concern” was negatively related to the AQ in females only. Moreover, the AQ was positively related to the SQ-R in females only. Lastly, the SQ-R and a number of the empathy scales were significantly associated: For example and surprisingly, the EQ correlated weakly and positively with the SQ-R in both male and female participants. The results from this study illustrate how standard self-report measures of empathy, autistic tendencies, and systemizing tendencies are associated with each other in a large sample not diagnosed with an autism spectrum disorder. Additionally, some potential gender-specific effects are revealed.

## Introduction

Empathy can be understood as an important concept contributing greatly toward successful human social interaction (1–3). However, despite empathy being a widely used term in science, as well as in everyday life, a consensus definition of the concept remains somewhat elusive [see, for example, the review and discussion of this topic in Refs. (4, 5)]. Nevertheless, there is some agreement that empathy comprises both affective (i.e., feeling similar emotions to another person) and cognitive components (i.e., understanding the feelings of another person) [for a summary covering many definitions of empathy, please see Ref. (4), and for examples, please see Refs. (6, 7)].

In line with the various definitions of empathy, a range of self-report measures assessing individual differences in empathy exist [e.g., Refs. (6–9)]. These questionnaires assess the degree of empathy on a continuum from low to high empathy. Two widely used measures in this area of research are the Interpersonal Reactivity Index (IRI) and the Empathy Quotient (EQ) (6, 8). The IRI assesses empathy using four distinct scales/dimensions of empathy labeled “Perspective Taking” (PeT), “Empathic Concern” (EmC), “Personal Distress” (PeD), and “Fantasy” (Fan). The PeT scale assesses the ability/tendency to take another’s perspective. The EmC scale assesses the extent to which someone feels warmth, but also concern for others. The PeD scale assesses self-oriented feelings of tension and worry in difficult social situations, or when someone else is hurt or in danger. Lastly, the Fan scale asks participants about their tendency to relate to fictional characters (8, 10). In most studies, it is claimed that the PeT and Fan scales measure cognitive aspects of empathy, whereas the EmC and PeD scales assess affective aspects of empathy. However, there is some controversy about this putative structure. For example, not all researchers agree that the Fan scale actually measures a facet of empathy (2, 3, 11–15). Moreover, there has been a suggestion that the PeD scale strongly overlaps with facets of the personality dimension Neuroticism (3). In contrast to the IRI, the EQ was originally developed to measure empathy as a unidimensional construct. The authors justify this decision by arguing that cognitive and affective components of empathy cannot be easily separated (6). Studies across various countries that have examined the associations between the scores of both self-report measures show that the EQ has the strongest and most consistent positive associations with the PeT and EmC scales of the IRI (2, 14, 16, 17).

Different versions of the EQ are often used in studies investigating empathy in the context of the neurodevelopmental condition of an autism spectrum disorder (ASD; see results reported below). ASD is/will be classified (including diagnostic criteria) and divided into various subtypes in the *International Classification of Diseases 11th Revision* (ICD-11) and the *Diagnostic and Statistical Manual of Mental Disorders, Fifth Edition* (DSM-5). It is—among other things—characterized by deficits in social functioning and communication (18, 19). According to the previous literature, samples of people diagnosed with an ASD show lower scores in empathy, as measured using different versions of the EQ, compared to control samples (20–24). However, autistic tendencies can also be studied on a continuum in the normal population. One instrument that assesses autistic tendencies using a dimensional approach is the Autism Spectrum Quotient (AQ) (21, 25, 26). As such, the AQ has been correlated with the EQ in previous studies (using a range of slightly different versions of the measures). Studies consistently report a negative association between the two measures in both control samples and samples of participants diagnosed with an ASD (6, 20, 21, 24, 27). To the best of our knowledge, however, there is only one study that has previously reported associations between the AQ and IRI scores. In this study, negative correlations between the AQ and the IRI scales PeT and EmC, and to a lesser extent Fan, were found. On the other hand, positive associations between the AQ score and the PeD scale were reported in these largely student samples from Germany and China (28).

Aside from reporting lower levels of empathy, samples of participants diagnosed with an ASD also show higher systemizing tendencies compared to control samples (20, 21, 24). These tendencies describe “the drive to analyze, understand, predict, control and construct rule-based systems” (p. 48) (21). Additionally, these tendencies might explain some of the characteristic symptoms of an ASD, such as repetitive behavioral patterns and problems with change (19, 29). A widely used self-report measure that assesses individual systemizing tendencies in the general population, as well as in people diagnosed with an ASD, is the systemizing quotient (SQ), and its revised version (SQ-R) (20, 21). Again, using a dimensional approach to measurement of this construct, correlations between autistic and systemizing tendencies have been shown to be positive for both control samples and those diagnosed with an ASD (20, 21, 24). This supports the idea that the key characteristics of an ASD can be observed in mild forms in participants sampled from the general population.

Although higher autistic tendencies are robustly associated with both lower empathy and higher systemizing tendencies, empathy and systemizing tendencies are not robustly correlated with each other. Correlations between scores in different EQ and SQ(-R) versions have been reported to lie between −0.06 and −0.21 in general population samples, and at around −0.29 in a sample of people diagnosed with an ASD (20, 21, 24, 30, 31). No study so far, however, seems to have reported associations between the IRI and the SQ-R.

Taken together, the aim of the present study was to investigate the relationships between empathy, autistic tendencies, and systemizing tendencies. Previous studies investigating these relationships typically used versions of the EQ, AQ, and SQ(-R). The findings previously shown with these scales should be replicated. In addition to this, the present study also sought to examine these relationships using the IRI, which, contrary to the EQ (which assesses empathy as unidimensional construct), assesses empathy as a multidimensional construct. In line with results from previous studies, we expected the following associations:

1. Positive correlations between the EQ and the IRI scales PeT and EmC
2. Negative correlations between the AQ and both the EQ and the IRI scales PeT and EmC
3. Positive correlations between the AQ and the SQ-R
4. Positive correlations between the AQ and the IRI PeD scale
5. No significant correlations between both the EQ and the IRI scales with the SQ-R^1^

## Materials and methods

### Registration

This study is registered at https://osf.io/q2arp/. Data will be made available upon reasonable request.

### Participants

Participants took part in an online study including various self-report questionnaire measures presented using the SurveyCoder-Tool (https://www.ckannen.com/). The data collection took place at Ulm University, Ulm, Germany. Therefore, most participants tended to be younger and were students. More specifically, 1,249 participants took part online in the present study, which is part of the Ulm Gene Brain Behavior Project (UGBBP). However, 11 participants were excluded due to missing data. Hence, 1,238 participants (373 males) were retained for subsequent analyses. One participant reported suffering from Asperger syndrome. This participant was excluded from further analyses, as he/she was identified as an outlier in terms of the AQ score.

More specifically, after calculating scores of all scales under investigation (see the paragraphs on Self-Report Measures), 140 participants were excluded due to their categorization as an outlier on at least one of the scales under investigation, or because of their reported age. All participants who scored lower than [25^th^-Quantil − (1.5x(75^th^-Quantil − 25^th^-Quantil))] or higher than [75^th^-Quantil + (1.5x(75^th^-Quantil − 25^th^-Quantil))] on at least one of the scales under investigation, or for age, were treated as outliers and excluded from further analyses (32). This is also the formula used in the Statistical Package for the Social Sciences (SPSS) statistics software of the International Business Machines Corporation (IBM) to detect outliers (unidimensionally) by means of the boxplot method. Results from the total sample, including those participants identified as outliers, are presented in the **Supplementary Material**. As can be seen there, the results of the analyses are similar whether the participants classified as outliers are included or not.

The mean age of the final sample of *N* = 1,098 participants (304 males) was 21.94 years (SD = 2.72 years; median = 21 years). The age range was 18–30 years.

All subjects gave electronic informed consent in accordance with the Declaration of Helsinki. The protocol was approved by the local ethics committee of Ulm University, Ulm, Germany.

It should be noted that as all participants are members of the UGBBP, the sample reported in this study partly overlaps with other samples derived from this project. For example, a previous study investigated a smaller subsample in relation to associations between oxytocin receptor genetics and the IRI and the AQ (28). Moreover, the IRI was also investigated in a recent experimental study on music perception in *n* = 160 participants from the UGBBP (33).

### Self-Report Measures

#### Interpersonal Reactivity Index

A German version of the Interpersonal Reactivity Index (IRI) was administered to measure empathy multidimensionally (8, 10). It consists of 28 items split into four scales. These are named “Perspective Taking” (PeT), “Empathic Concern” (EmC), “Personal Distress” (PeD), and “Fantasy” (Fan). Each scale consists of seven items. No total score across all items is calculated. All items are answered on a five-point Likert scale from “0” to “4”. The internal consistencies (using Cronbach’s alpha) of the four scales in the present sample were as follows: PeT: .78, EmC: .82, PeD: .76, Fan: .81.

#### Empathy Quotient

A German version of the 60-item Empathy Quotient (EQ) was used in the study to measure empathy unidimensionally (6). The items of this questionnaire are answered on a four-point Likert scale. The answer to each item is transformed into either “0” (for two of the four responses indicating nonempathic tendencies), “1,” or “2,” with higher values indicating higher empathy. The scores for 40 of the items are then summed to create a total scale score. It should be noted that it is also possible to calculate several subscales from the EQ. However, such subscales were not originally intended by the authors (6). Additionally, details about such subscales remain debatable (16, 17, 34–36). The present study therefore focused on the total scale. Its internal consistency (using Cronbach’s alpha) in the present sample was .88.

#### Autism Spectrum Quotient

A German version of the Autism Spectrum Quotient (AQ) was used to measure autistic tendencies in the current study (25, 26). It consists of 50 items answered on a four-point Likert scale. The answer to each item is transformed into a “1” for more autistic-type responses, and a “0” for nonautistic-type responses. From this, a total score, as well as scores for several subscales, can be calculated [see Refs. (26, 37) for different approaches to splitting the AQ into subscales]. The current study focused only on the total scale score. Its internal consistency (using Cronbach’s alpha) was .73 in the present sample.

#### Systemizing Quotient–Revised

The German version of the Systemizing Quotient–Revised (SQ-R) was used to measure systemizing tendencies (21) (German version available from: http://docs.autismresearchcentre.com/tests/SQ_German.pdf). It consists of 75 items, which are answered on a four-point Likert scale. The answer to each item is transformed into either “0” (for two of the four answer options indicating nonsystemizing tendencies), “1,” or “2,” with higher scores indicating stronger systemizing tendencies. From this, a total score is calculated by summing across the items. The internal consistency (using Cronbach’s alpha) of this scale was .85 in the present sample.

### Statistical Analyses

All analyses were implemented using SPSS statistics version 24.

First, the distributions of the scales under investigation were checked for a normal distribution. The statistical tests (Kolmogorov–Smirnov and Shapiro–Wilk) indicated significant deviations from the normal distribution for all scales. This is most likely due to the large number of participants in the present study. Therefore, the skewness and kurtosis of all distributions were additionally checked. For all of the scales under investigation, as well as age, the skewness and kurtosis were smaller than +/−1. Hence, in line with the rules of thumb suggested by Miles and Shevlin, normality could be assumed (38). Inspecting the histograms of all scales also led to the conclusion that an approximate normal distribution could be assumed.

Following this, the associations between the scales of all of the self-report measures included in the study, along with age, were calculated using Pearson correlations. Next, differences across gender were investigated using *t*-tests (if necessary, Welch’s *t*-tests were used and reported).

Finally, associations between the scales were calculated using partial Pearson correlations, controlling for age (see significant associations with age). These analyses were implemented in the total sample (*N* = 1,098), as well as split by gender. This procedure was chosen given the unequal gender distribution and the differences in the mean scores of the self-report measures across males and females. The correlations were compared across males and females using Fisher’s *z*-tests (http://www.markenkunde.de/korrleation_tool/markenkunde_corrcomparer1_0.xls).

We present correlational analyses between the self-report measures instead of, for example, regression analyses in the main manuscript in order to report “unbiased” associations (i.e., not controlling for potential overlaps between the self-report measures). However, as an additional and exploratory analysis, a regression model to predict the AQ score is also presented in the **Supplementary Material**.

All results were evaluated for significance using two-tailed tests.

## Results

### Associations With Age and Gender

Age correlated significantly with the IRI scales EmC (*r* = −0.10, *p* < 0.001), PeD (*r* = −0.12, *p* < 0.001), and Fan (*r* = −0.17, *p* < 0.001), and with the EQ (*r* = −0.11, *p* < 0.001). The *p*-values for all other correlations with age were >0.247. Age was therefore controlled for in further analyses.

Significant differences across gender were found for all of the scales under investigation. For descriptive statistics and results of the *t*-tests, please see **Table 1**. Females scored higher on all empathy-related scales, whereas males scored higher on the AQ and SQ-R.

**Table 1:** Descriptive statistics for all scales under investigation and *t*-tests for gender differences.

	Total sample (*N* = 1,098)		Males (*n* = 304)		Females (*n* = 794)		*t*-test		Hedge’s g
	M	SD	M	SD	M	SD	*t*(df)	*p*	
IRI PeT	17.29	4.29	16.55	4.38	17.57	4.23	*t*(1096) = −3.53	<0.001	−0.238
IRI EmC	19.07	4.51	16.38	4.47	20.09	4.09	*t*(507.47) = −12.61	<0.001	−0.886
IRI PeD	13.50	4.29	11.24	3.78	14.37	4.16	*t*(599.19) = −11.93	<0.001	−0.772
IRI Fan	18.67	5.04	16.38	5.04	19.55	4.76	*t*(1096) = −9.74	<0.001	−0.657
EQ	42.18	10.60	36.34	9.75	44.42	10.06	*t*(1096) = −12.01	<0.001	−0.810
AQ	16.47	5.66	18.01	5.54	15.89	5.59	*t*(1096) = 5.65	<0.001	0.381
SQ-R	51.20	14.80	56.59	14.64	49.13	14.35	*t*(1096) = 7.66	<0.001	0.517

### Correlations Between the Self-Report Measures

Correlations between the self-report measures in the total sample are presented in **Table 2** (without any correction for multiple testing). After manually applying a Bonferroni correction for multiple testing (alpha = 0.05/21 = 0.0024; divided by 21 because 21 correlations were calculated), the following results with regard to the hypotheses remained significant: Partly in line with the first hypothesis, the EQ correlated significantly and positively with the IRI scales PeT, EmC, but also Fan. The EQ and the IRI scales PeT and EmC correlated significantly and negatively with the AQ. This is in line with the second hypothesis. The AQ correlated significantly and positively with the SQ-R and with the IRI scale PeD, which supports the third and fourth hypotheses. In relation to the fifth hypothesis, the SQ-R showed (mostly) weak correlations with the empathy measures. Only its negative correlation with PeD remained significant.

**Table 2:** Partial correlations between all scales under investigation in the full sample.

	IRI PeT	IRI EmC	IRI PeD	IRI Fan	EQ	AQ	SQ-R
IRI PeT							
IRI EmC	0.40***						
IRI PeD	−0.03	0.30***					
IRI Fan	0.20***	0.39***	0.22***				
EQ	0.47***	0.61***	0.04	0.33***			
AQ	−0.23***	−0.22***	0.26***	−0.08**	−0.43***		
SQ-R	0.09**	−0.01	−0.18***	0.09**	0.07*	0.18***	

N = 1,098. All correlations are corrected for age. ***p < 0.001, **p < 0.01, *p < 0.05 (two-tailed). Of the significant (*p < 0.05) correlations reported in this table, those between IRI PeT and SQ-R (p = 0.003), between IRI Fan and AQ (p = 0.005), between IRI Fan and SQ-R (p = 0.003), and between EQ and SQ-R (p = 0.028) would not remain significant after manually applying a Bonferroni correction for multiple testing (alpha = 0.05/21 = 0.0024).

The correlations between the self-report measures for males and females separately are presented in **Table 3** (without any correction for multiple testing). After manually applying a Bonferroni correction for multiple testing (alpha = 0.05/21 = 0.0024), the following significant correlations with regard to the hypotheses remained significant: Within the male and female sample, the EQ was significantly and positively related to the PeT and the EmC scales of the IRI. This supports the first hypothesis. The EQ was also significantly and positively related to the Fan scale of the IRI in both samples. The AQ was significantly and negatively related to the EQ and the IRI scales PeT and EmC for females, but for males, only the negative relationships between the AQ and both the EQ and the IRI scale PeT were significant. Hence, the second hypothesis is only fully supported for females. Regarding the third hypothesis, the AQ was significantly and positively related to the SQ-R in the female sample only. Moreover, the AQ was significantly and positively related to the IRI scale PeD for both males and females, which supports the fourth hypothesis. Contrary to the fifth hypothesis, in the male and female samples, the SQ-R correlated weakly but significantly with the EQ (positively) and some of the IRI scales (negatively with the IRI scale PeD for males; positively with the IRI scale Fan for females).

**Table 3:** Partial correlations between all scales under investigation for males and females separately.

	IRI PeT	IRI EmC	IRI PeD	IRI Fan	EQ	AQ	SQ-R
IRI PeT		0.41***	0.04	0.25***	0.51***	−0.20***	0.15**
IRI EmC	0.39***		0.32***	0.35***	0.55***	−0.07	0.14*
IRI PeD	−0.10**	0.17***		0.22***	−0.03	0.32***	−0.20***
IRI Fan	0.15***	0.32***	0.12***		0.30***	−0.02	0.09
EQ	0.45***	0.57***	−0.09*	0.26***		−0.39***	0.23***
AQ	−0.22***	−0.22***	0.33***	−0.05	−0.41***		0.07
SQ-R	0.10**	0.05	−0.09*	0.19***	0.12***	0.18***	

Results for the male participants (n = 304) are presented above the diagonal. Results for the female participants (n = 794) are presented below the diagonal. All correlations are corrected for age. ***p < 0.001, **p < 0.01, *p < 0.05 (two-tailed). Of the significant (*p < 0.05) correlations, the correlations between IRI PeT and SQ-R (p = 0.008) and between IRI EmC and SQ-R (p = 0.017) for males, and between IRI PeT and SQ-R (p = 0.004), between IRI PeD and SQ-R (p = 0.012), between IRI PeD and EQ (p = 0.013), and between IRI PeT and PeD (p = 0.004) for females would not remain significant after manually applying a Bonferroni correction for multiple testing (alpha = 0.05/21 = 0.0024).

Of note, only a few of the correlations between males and females differed significantly. These were the correlations between the AQ and the IRI scale EmC (*z* = 2.18, sigma = 0.068, *p* = 0.029; higher negative correlation in the female sample as compared to the male sample), between the IRI scales PeT and PeD (*z* = 2.07, sigma = 0.068, *p* = 0.038; negative correlation in the female sample and weakly positive correlation in the male sample), and between the IRI scales EmC and PeD (*z* = 2.31, sigma = 0.068, *p* = 0.021; higher positive correlation in the male sample as compared to the female sample).

Lastly, to further elucidate the correlations between the AQ and the SQ-R, especially the nonsignificant result found for males, the relationships between the AQ subscales and the SQ-R were also examined for males and females separately. These results are presented in the **Supplementary Material** as additional *post hoc* analyses.

## Discussion

The present study sought to investigate the relationships between standard self-report measures assessing empathy, autistic tendencies, and systemizing tendencies in a large sample of participants not diagnosed with an ASD. More specifically, the current study sought to extend the existing literature that has examined these links by considering the relationships between autistic and systemizing tendencies and a unidimensional measure of empathy, the EQ, as well as a multidimensional measure of empathy, the IRI.

Firstly, gender differences typically found in healthy/control samples for empathy (i.e., females > males) and for autistic and systemizing tendencies (i.e., males > females) were also found in the present study [e.g., Refs (6, 8, 20, 21, 24, 26, 30)].

Consistent with most of the literature, the EQ correlated most strongly with the IRI scales PeT and EmC for both males and females (2, 14, 16, 17). With a correlation of around .50, these associations can be considered a large effect size (39). However, it should be noted that the Fan scale of the IRI was also significantly and positively related to the EQ, albeit more weakly, in males and females. This is also in line with earlier studies (2, 16). These effect sizes can be considered medium in magnitude (39), and they might indicate that the Fan scale of the IRI is indeed associated with empathic processes [see also Ref. (2)].

The AQ was significantly and negatively related to the EQ and the IRI scale PeT for both males and females with medium effect sizes (39). Additionally, the AQ was negatively related to the IRI scale EmC in the female sample only. Hence, for the female participants, the results of the present study are similar to the results from the study by Montag and colleagues on which the second hypothesis in the present study was partly based (28). It should be noted, though, that the samples for these studies overlap, as described in the “Participants” section above. The results from the present study also suggest that, for males, lower cognitive empathy (IRI scale PeT), but not affective empathy (IRI scale EmC), might be associated with higher autistic tendencies, and ultimately an ASD. In line with this, results from earlier studies also indicate that specifically cognitive empathy (using the IRI, among other instruments) is lower for those with an ASD. On the other hand, the scores for affective empathy in groups diagnosed with an ASD are typically similar to the scores in the respective control group [e.g., Refs. (40, 41)]. It should be noted that the samples from these studies consisted mostly of males, underlining the idea that these differential associations are relatively specific to males. However, for the females in the present study, no conclusions can be drawn regarding differential associations of autistic tendencies with affective (IRI scales EmC and PeD) and cognitive (IRI scales PeT and Fan) aspects of empathy.

The AQ was significantly and positively related to the SQ-R for females, but not for males; for females, the effect size of the correlation between these variables (*r* = 0.18) could only be considered small to medium (39). Investigating the scatterplots of the associations between the AQ and the SQ-R for males revealed that the nonsignificant findings in this sample could not be explained by potential outliers or limited variance. Nonetheless, the nonsignificant, near-zero correlation for males can potentially be explained by the different correlations between the SQ-R and the AQ subscales (see **Supplementary Material**). More specifically, after implementing a correction for multiple testing, in males, the SQ-R was only significantly and positively related to the AQ subscale “Attention to Detail”. When only considering the total AQ score, this effect appears to have been obscured by the nonsignificant, sometimes negative, associations of the SQ-R with other subscales of the AQ in the male participants. It should be noted, however, that the “Attention to Detail” subscale of the AQ and systemizing tendencies, as measured by the SQ-R, overlap in their content (21, 25, 26). Moreover, the applicability of the AQ subscales to neurotypical samples remains questionable (see, for example, low internal consistencies and test–retest reliabilities, as well as the finding that neurotypical participants score higher on “Attention Switching” and “Attention to Detail” compared to the other scales, which is not necessarily the case for patients) (25, 26, 42). In sum, the associations between the AQ and SQ-R in male-only samples not diagnosed with an ASD should be investigated in more detail in future studies.

The AQ was significantly and positively related to the IRI scale PeD in males and females. Hence, higher autistic tendencies seem to be associated with higher feelings of anxiety and tension in interpersonal situations (10). This linear association fits with the social and communicative problems often observed in an ASD according to the diagnostic criteria (18, 19).

Lastly, the SQ-R was significantly and positively related to the EQ in both males and females. In the female sample, the effect size was small; however, in the male sample, the effect size was small to medium (39). Moreover, in the male sample, this correlation was even higher than the correlation between the SQ-R and the AQ, but the difference was not significant (*t* = 1.80, *p* = .073). Some of the IRI scales were also significantly related to the SQ-R depending on the sample (males versus females). This is in contrast to previous results and assumptions indicating that empathy and systemizing tendencies are (weakly) negatively related and therefore potentially distinct and independent phenomena (21, 24, 30). Given these previous results, it is difficult to explain the positive correlations found in the current study. It may be possible that the positive correlations reflect a common cognitive component shared by the empathy measures and the SQ-R, as mostly the IRI scales assessing cognitive empathy were more strongly positively correlated with the SQ-R, as compared to the scales assessing affective empathy. Nevertheless, we do not want to overinterpret these findings, as the effect sizes were not particularly high.

Some limitations of the present study should be mentioned. Firstly, the sample consisted of participants not diagnosed with an ASD, limiting the generalizability of the results from the study. Moreover, other demographic variables, and clinical symptom severity for other disorders, such as depression or anxiety, might also be worth considering in relation to ASD.

In regard to the latter point, participants also completed the BDI-II (Beck Depression Inventory-II; assessing depression symptoms) and the Affective Neuroscience Personality Scales (ANPS), including its FEAR scale (assessing facets of anxiety using a trait approach) (43–45). The results presented in **Tables 2** and **3** did not, however, change substantially if the BDI-II score or the FEAR scale was included as a control variable alongside age. Further information on these analyses is given in the **Supplementary Material**.

Next, the sole use of self-report questionnaires as measures for the constructs could be criticized. Clearly, this methodology has several disadvantages (46). The specific questionnaires used in the study might be criticized too. For example, the AQ (including all 50 items) was found to show high sensitivity, but low specificity, with clinical ASD diagnosis as a criterion, and overall did not predict clinical ASD diagnosis very well in a sample of participants suspected of a potential ASD diagnosis. Also, the correlations between the AQ score, and current ASD behaviors and reported early-life ASD symptoms, were weak in this sample (47). Moreover, in this paper, it is also hypothesized that the AQ score might be positively influenced by anxiety (hence, not only explicit ASD symptoms), again pointing toward the importance of assessing markers of other psychiatric disorders. In the current study, we can only provide information about the FEAR score, which is assessing trait anxiety, but not anxiety disorder symptoms (as noted above). Another study found that items of the AQ often correlated more strongly with scales assessing constructs such as psychological distress, sleepiness, quality of life, psychoticism, or alcoholism than with their own AQ scale. It was also found that the total AQ score correlated positively with psychological distress and psychoticism in controls and patients (48). However, criticizing the self-report measures was not within the scope of the present study; rather, the aim of the present study was to investigate relationships between these self-report measures. This is clearly important given their widespread use in research and applied contexts. Moreover, self-report measures also have several advantages. They are easy to use and cheap, especially if large sample sizes are required, and the interpretation of the results is straightforward. Most importantly, to assess subjective experiences in relation to latent traits, such as empathy, autistic tendencies, or systemizing tendencies, we need to ask the individuals themselves. Additionally, we aimed to provide further insights into associations between self-reported empathy (using unidimensional and multidimensional measures), autistic tendencies, and systemizing tendencies using a dimensional approach to measurement. Newer measures also exist that assess empathy in relation to specific emotions (49). These measures should also be investigated in relation to the widely used measures covered in the present study.

In conclusion, the results from this study provide insights into the relationship between empathy, autistic tendencies, and systemizing tendencies in a large, mostly student, sample not diagnosed with an ASD. The results generally support the notion that autistic tendencies are negatively related to empathy, but positively related to systemizing tendencies, although in males the association between autistic and systemizing tendencies was weak and nonsignificant. Therefore, the results suggest that future studies should report findings with these measures separately for males and females, and also support the notion that future studies should focus on facets of autistic tendencies and empathy to examine these relations in more fine-grained detail.

## Ethics Statement

All subjects gave electronic informed consent in accordance with the Declaration of Helsinki. The protocol was approved by the local ethics committee of Ulm University, Ulm, Germany.

## Author Contributions

CS and CM planned and implemented the study and collected the data. CS implemented the statistical analyses and wrote the manuscript. CM worked over the manuscript to improve it. AC gave helpful advice and also worked over the manuscript to improve it.

## Funding

The position of CM is funded by a Heisenberg grant awarded to him by the German Research Foundation (DFG, MO2363/3-2).

## Conflict of Interest Statement

The authors declare that the research was conducted in the absence of any commercial or financial relationships that could be construed as a potential conflict of interest.

## Footnotes

1 No significant associations were expected, because especially in control samples (not diagnosed with an ASD) the associations found in previous literature were of rather low effect size.
