# Supplementary material for: Empathy, Autistic Tendencies, and Systemizing Tendencies—Relationships Between Standard Self-Report Measures
Source: Front Psychiatry. 2019 May 10;10:307. doi: 10.3389/fpsyt.2019.00307 (PMC6522547; doi:10.3389/fpsyt.2019.00307)
Supplement: Supplementary file 1 [file DataSheet_1.docx]

**Empathy, Autistic Tendencies, and Systemizing Tendencies – Relationships Between Standard Self-Report Measures**

Cornelia Sindermann, Andrew Cooper, Christian Montag

***Supplementary Material***

**1 Results including the outliers**

As mentioned in the main manuscript, 140 participants were excluded due to their categorization as outliers prior to the final data analysis. For completeness, we also report the main results calculated based on the complete sample (including outliers; *N*=1,238 participants (373 males)) here. These results are presented in Supplementary Tables 1, 2 and 3.

Of note, age was not normally distributed (skewness: 3.68 (SE=0.07), kurtosis: 15.84 (SE=0.14)) in this sample. However, we still report the partial Pearson correlations, corrected for age, in the Supplementary Tables 2 and 3 for better comparability with the results of the main manuscript.

Supplementary Table 1.
Descriptive statistics for all scales under investigation and *t*-tests for gender differences.

|  | **Total sample (***N***=1,238)** | | **Males (***n***=373)** | | **Females (***n***=865)** | | ***t*-test** | |
| --- | --- | --- | --- | --- | --- | --- | --- | --- |
|  | M | SD | M | SD | M | SD | *t*(df) | *p* |
| IRI PeT | 17.25 | 4.37 | 16.43 | 4.51 | 17.60 | 4.26 | *t*(1236)=-4.35 | <0.001 |
| IRI EmC | 18.77 | 4.98 | 15.83 | 5.17 | 20.05 | 4.32 | *t*(605.17)=-13.81 | <0.001 |
| IRI PeD | 13.22 | 4.47 | 10.84 | 4.05 | 14.24 | 4.24 | *t*(1236)=-13.13 | <0.001 |
| IRI Fan | 18.33 | 5.30 | 15.98 | 5.30 | 19.34 | 4.97 | *t*(1236)=-10.72 | <0.001 |
| EQ | 41.93 | 11.10 | 36.21 | 10.42 | 44.40 | 10.46 | *t*(1236)=-12.65 | <0.001 |
| AQ | 16.66 | 5.90 | 18.24 | 5.95 | 15.97 | 5.75 | *t*(1236)=6.29 | <0.001 |
| SQ-R | 52.33 | 16.15 | 58.53 | 16.75 | 49.65 | 15.13 | *t*(645.46)=8.81 | <0.001 |

Supplementary Table 2.
Partial correlations between all scales under investigation in the full sample.

|  | **IRI PeT** | **IRI EmC** | **IRI PeD** | **IRI Fan** | **EQ** | **AQ** | **SQ-R** |
| --- | --- | --- | --- | --- | --- | --- | --- |
| IRI PeT |  |  |  |  |  |  |  |
| IRI EmC | 0.42*** |  |  |  |  |  |  |
| IRI PeD | -0.02 | 0.35*** |  |  |  |  |  |
| IRI Fan | 0.22*** | 0.44*** | 0.27*** |  |  |  |  |
| EQ | 0.50*** | 0.63*** | 0.06* | 0.36*** |  |  |  |
| AQ | -0.26*** | -0.25*** | 0.21*** | -0.11*** | -0.45*** |  |  |
| SQ-R | 0.07** | -0.08** | -0.22*** | 0.04 | 0.05 | 0.23*** |  |

Note: *N*=1,238. All correlations are corrected for age. ****p*<0.001, ***p*<0.01, **p*<0.05 (two-tailed). Of the significant (**p*<0.05) correlations reported in this table, those between IRI PeT and SQ-R (*p*=0.010), between IRI EmC and SQ-R (*p*=0.003), and between IRI PeD and EQ (*p*=0.046) would not remain significant after manually applying a Bonferroni correction for multiple testing (alpha=0.05/21=0.0024).

Supplementary Table 3.
Partial correlations between all scales under investigation for males and females separately.

|  | **IRI PeT** | **IRI EmC** | **IRI PeD** | **IRI Fan** | **EQ** | **AQ** | **SQ-R** |
| --- | --- | --- | --- | --- | --- | --- | --- |
| IRI PeT |  | 0.41*** | 0.01 | 0.24*** | 0.55*** | -0.26*** | 0.12* |
| IRI EmC | 0.41*** |  | 0.35*** | 0.37*** | 0.53*** | -0.12* | -0.01 |
| IRI PeD | -0.10** | 0.20*** |  | 0.25*** | -0.05 | 0.31*** | -0.22*** |
| IRI Fan | 0.18*** | 0.37*** | 0.16*** |  | 0.29*** | -0.04 | 0.09 |
| EQ | 0.47*** | 0.59*** | -0.07* | 0.30*** |  | -0.44*** | 0.20*** |
| AQ | -0.23*** | -0.24*** | 0.29*** | -0.08* | -0.42*** |  | 0.13* |
| SQ-R | 0.11** | 0.03 | -0.11** | 0.14*** | 0.12*** | 0.22*** |  |

Note: Results for the male participants (*n*=373) are presented above the diagonal. Results for the female participants (*n*=865) are presented below the diagonal. All correlations are corrected for age. ****p*<0.001, ***p*<0.01, **p*<0.05 (two-tailed). Of the significant (**p*<0.05) correlations, the correlations between IRI PeT and SQ-R (*p*=0.021), between IRI EmC and AQ (*p*=0.016) as well as between AQ and SQ-R (*p*=0.011) for males, and between IRI PeT and PeD (*p*=0.004), between IRI PeD and EQ (*p*=0.029), and between IRI Fan and the AQ (*p*=0.020) for females would not remain significant after manually applying a Bonferroni correction for multiple testing (alpha=0.05/21=0.0024).

**2 Regression analyses to predict the AQ**

To further investigate the associations between the AQ and the other scales, we also implemented a multiple, hierarchical, stepwise (forward) regression analysis. Age and gender were included in the first block. The IRI scales, the EQ and the SQ-R score were included in a second block, and the gender by IRI, EQ, SQ-R interaction scores in a third block. The final model, including only the significant predictors, is presented in Supplementary Table 4.

Supplementary Table 4.
Regression analyses to predict the AQ score.

|  | **β** | ***t*** | ***p*** |
| --- | --- | --- | --- |
| constant |  | 17.73 | <0.001 |
| Gender | -0.07 | -2.50 | 0.012 |
| EQ | -0.45 | -16.79 | <0.001 |
| IRI PeD | 0.34 | 13.07 | <0.001 |
| SQ-R | 0.25 | 9.89 | <0.001 |

Note: *R*^2^ of the final model was 0.34.

**3 Post-Hoc analyses for the male participants**

Due to the surprising, non-significant and near-zero correlation between the AQ and the SQ-R score for the male sample reported in the main manuscript, this association was investigated in more detail. Therefore, the AQ was split into the originally proposed sub-scales (poor) “Social Skill”, (poor) “Attention Switching”, “Attention to Detail”, (poor) “Communication”, and (poor) “Imagination” (1, 2). Partial Pearson correlations (corrected for age) between the SQ-R and these sub-scales for the male sample revealed that the SQ-R was significantly associated with the sub-scales “Social Skill” (*r*=-0.15, *p*=0.011), “Attention to Detail” (*r*=0.41, *p*<0.001), and “Communication” (*r*=-0.13, *p*=0.028). On the other hand, it was not significantly associated with the sub-scales “Attention Switching” (*r*=0.05, *p*=0.409) and “Imagination” (*r*=0.01, *p*=0.910). Additionally, the negative correlations with the sub-scales “Social Skill” and “Communication” would not hold after manually applying a Bonferroni correction (e.g. alpha=0.05/5=0.01; divided by 5 for the five sub-scales of the AQ). Hence, these latter correlations should be interpreted cautiously. As an additional note: when implementing stepwise regression analyses to predict the SQ-R score by the AQ sub-scales, the AQ sub-scales “Attention to Detail” (β=0.41, *t*=7.83, *p*<0.001) and “Communication” (β=-0.11, *t*=-2.20, *p*=0.029) are significant predictors in the final model (*R*^2^=0.18).

**4 Post-Hoc analyses for the female participants**

For completeness, we also examined the associations between the AQ sub-scales and the SQ-R for the female sample. Again, Partial Pearson correlations (corrected for age) were used for the analyses. The SQ-R was significantly associated with the sub-scales “Attention Switching“ (*r*=0.07, *p*=0.048), “Attention to Detail” (*r*=0.49, *p*<0.001), and “Imagination” (*r*=-0.07, *p*=0.048). On the other hand, the SQ-R was not significantly associated with the sub-scales “Social Skill” (*r*=0.01, *p*=0.737) and “Communication” *(r*=-0.06, *p*=0.090). Only the association with the sub-scale “Attention to Detail” would remain significant after manually applying a Bonferroni correction (e.g. alpha=0.05/5=0.01; divided by 5 for the five sub-scales of the AQ). When implementing stepwise regression analyses to predict the SQ-R score by the AQ sub-scales, the AQ sub-scales “Attention to Detail” (β=0.49, *t*=15.78, *p*<0.001), “Imagination” (β=-0.08, *t*=-2.65, *p*=0.008), and “Attention Switching” (β=0.06, *t*=1.98, *p*=0.048) are significant predictors in the final model (*R*^2^=0.25).

**5 Controlling for the BDI-II score and the ANPS FEAR scale when investigating the correlations between the self-report measures IRI, EQ, AQ, SQ-R**

As mentioned in the main manuscript, considering symptom severity for disorders, such as depression or anxiety, might also be worth considering in relation to ASD. In this regard, we can report that the sample showed a mean BDI-II score of M=8.37 (SD=7.41), and a Median of 6.00 (3). Most of the participants had a score lower than 13, indicating no current depression (*n*=866, 79%). The BDI-II score correlated significantly with the AQ score (*r*=0.34, *p*<0.001), IRI PeT (*r*=-0.07, *p*=0.014), IRI PeD (*r*=0.42, *p*<0.001), IRI Fan (*r*=0.09, *p*=0.002), and with the EQ (*r*=-0.13, *p*<0.001) in the total sample (all correlations corrected for age). The results presented in Tables 2 and 3 in the main manuscript did not, however, change substantially if the BDI-II score was included as a control variable alongside age. Participants also completed the Affective Neuroscience Personality Scales (ANPS), including its FEAR scale, which assesses anxiety as a trait rather than assessing anxiety disorder symptom severity (*n*=1,097; one participant provided incomplete data regarding the ANPS) (4, 5). This scale correlated significantly with the AQ (*r*=0.33, *p*<0.001), IRI PeT (*r*=-0.06, *p*=0.048), IRI EmC (*r*=0.21, *p*<0.001), IRI PeD (*r*=0.63, *p*<0.001), IRI Fan (*r*=0.22, *p*<0.001) and the SQ-R (*r*=-0.11, *p*<0.001) in the total sample (all correlations corrected for age). As with the BDI-II, the results presented in Tables 2 and 3 in the main manuscript did not change substantially if the ANPS FEAR score was included as a control variable alongside age. It should be noted, however, that the correlations between IRI PeD and the AQ turned out to be lower (i. e. *r*=0.07, *p*=0.025 in the total sample, *r*=0.16, *p*=0.005 in males, *r*=0.11, *p*<0.001 in females) when corrected for age and FEAR score. This is most likely due to the high intercorrelation between IRI PeD and the FEAR scale.

**References**

(1) Baron-Cohen S, Wheelwright S, Skinner R, Martin J, Clubley E. The autism-spectrum quotient (AQ): evidence from asperger syndrome/high-functioning autism, males and females, scientists and mathematicians. J Autism Dev Disord (2001) 31(1):5-17. https://doi.org/10.1023/A:1005653411471

(2) Baron-Cohen S, Hoekstra RA, Knickmeyer R, Wheelwright S. The autism-spectrum quotient (AQ) - adolescent version. J Autism Dev Disord (2006) 36(3):343-50. https://doi.org/10.1007/s10803-006-0073-6

(3) Beck AT, Steer RA, Brown GK. Beck Depression Inventory-II. San Antonio, TX: Psychological Corp. (1996).

(4) Davis KL, Panksepp J, Normansell L. The affective neuroscience personality scales: Normative data and implications. Neuropsychoanalysis (2003) 5(1):57-69. https://doi.org/10.1080/15294145.2003.10773410

(5) Reuter M, Panksepp J, Davis K, Montag C. Affective neuroscience personality scales (ANPS)–Deutsche Version, 1st ed. Göttingen: Hogrefe-Verlag (2017).
